# The Influence of Substituents in Phosphazene Catalyst-Flame Retardant on the Thermochemistry of Benzoxazine Curing

**DOI:** 10.3390/polym13183111

**Published:** 2021-09-15

**Authors:** Natalia V. Bornosuz, Roman F. Korotkov, Alexander A. Kolenchenko, Alexey V. Shapagin, Alexey V. Orlov, Irina Yu. Gorbunova, Vyacheslav V. Kireev, Igor S. Sirotin

**Affiliations:** 1 Faculty of Petrochemistry and Polymer Materials, Mendeleev University of Chemical Technology, 125047 Moscow, Russia; bornosuz@muctr.ru (N.V.B.); ro.korotkov@muctr.ru (R.F.K.); kolenchenkoalex@muctr.ru (A.A.K.); alexeyorlovvladimirovich3829@gmail.com (A.V.O.); igorbunova@muctr.ru (I.Y.G.); kireev@muctr.ru (V.V.K.); 2 Frumkin Institute of Physical Chemistry and Electrochemistry Russian Academy of Sciences (IPCE RAS), 31, Bld. 4 Leninsky Prospect, 119071 Moscow, Russia; shapagin@mail.ru

**Keywords:** benzoxazines cure, phosphazenes, flame retardant, catalysis, thermal analysis

## Abstract

This work is devoted to the influence of phosphazene modifiers with different substituents on the curing process, thermal properties and flammability of benzoxazine resin. Novel catalysts with m-toluidine substituents were introduced. The catalytic activity of studied phosphazene compounds decreased in the row: hexachlorocyclotriphosphazene (HCP) > tetra m-toluidine substituted phosphazene PN-mt (4) > hexa m-toluidine substituted phosphazene PN-mt (6) > hexaphenoxycyclotriphosphazene (HPP), where HPP is totally inactive. Two types of catalysis: basic and acid were proposed. A brief study of resulting properties of polybenzoxazines was presented. The addition of any studied modifier caused the decrease of glass transition temperature and thermal stability of polymers. The morphology of cured compositions was characterized by matrix-dispersion phase structure. All phosphazene containing polybenzoxazines demonstrated the improved flame resistance.

## 1. Introduction

Polymer composite materials (PCM) have become ingrained in our everyday lives. They have the advantages of low specific gravity combined with high strength, moisture and chemical resistance, radio transparency, excellent dielectric properties, durability, etc. Because of these properties PCM came into common use in automotive and shipbuilding industries, aircraft, sports, medical applications, and many other fields. However, technological progress and economic development have helped scientists improve the quality of PCM by modifying binder and filler. As one of the main disadvantages of both polymers and composites based on them is their high flammability due to the organic nature of the matrix, which limits wider application of PMC, coke-forming phenolic or polybenzoxazine matrices [1] or epoxy and polyester binders modified with flame retardants are usually used in transport interior industry [2,3]. However, the introduction, for instance, of phosphorus-containing flame retardants, as a rule, leads to a significant decrease in mechanical properties and heat resistance of the polymer due to the absence of compatibility between matrix and flame retardant resulting in a two-phase system.

**Figure 1:**
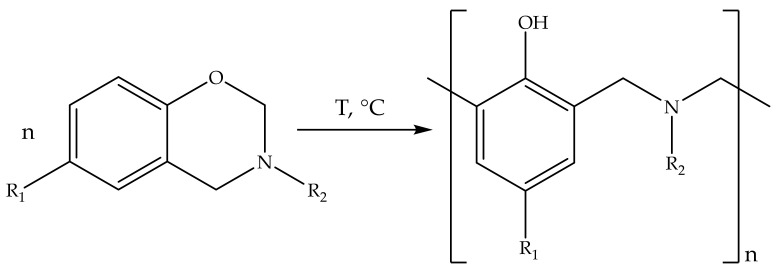
Formation of polybenzoxazine network by thermal curing process.

Our object of study is benzoxazine binders for PCM. It is a novel class of monomers commercialized in 2008. For now, many corporations such as Huntsman Advanced Materials and Henkel have branded benzoxazine binders and prepregs. Benzoxazine monomers have the feature of thermal self-curing (Figure 1) that could be proceeded by different mechanisms proposed in the literature [4]. Polybenzoxazines are characterized by a number of beneficial properties, including high mechanicals, dimensional stability and heat resistance, near-zero curing shrinkage, and low moisture absorption [5,6]. Some polybenzoxazines are completely nonflammable, for instance, ones based on 4,4′-diaminodiphenylmethane (P-d) and 3,3′-dichloro-4,4′-diaminodiphenylmethane (P-q) with phenol [7]. However, most of them have just reduced flammability reaching V-1/V-2 category by UL-94 tests. Another common problem of benzoxazine is high curing temperature, and therefor low energy efficiency, that limits their use in different technologies. The main way to solve this problem is to use the catalysts [8,9,10,11,12] such as phenols, strong acids [9], carboxylic acids and Lewis acids for the homopolymerization of benzoxazines. The base catalysts such as amines [13], imidazoles [14], and organophosphorus compounds, which exhibit a weaker catalyzing effect in comparison with acidic ones, are also applicable.

Thus, these are two problems of benzoxazine that our scientific group are to cope with: flammability and catalysis. Pursuing this complex goal phosphazene-based compounds may become widespread.

Phosphazenes are a unique class of heterocyclic compounds, discovered by Liebig, Wöhler, and Rose in 1834. The unique features of phosphazenes are their outstanding flexible molecular design and a wide range of homologous cyclo- and linear phosphazenes applied in many different fields. A lot of functionalized epoxy resins, benzoxazines, and amine-based curing agents with phosphazene core are proposed in the literature [6,15,16,17,18,19,20]. Cyclophosphazenes, owing to their flexible design, have the possibility of replacing chlorine atoms in hexachlorocyclotriphosphazenes with practically any substituents that in fact determine the properties of the resultant compound. They also may form numerous complexes. That is why scientists often use phosphazenes as a base for catalysts, extractants, and curing agents. Moreover, due to their inorganic nature they take on market as flame retardants [3]. The synergistic effect of phosphorus and nitrogen atoms enclosed in an unsaturated skeleton contributes to enhanced flame resistance [21,22] in the process of thermooxidative destruction (combustion). The mechanism of the synergistic effect in phosphazenes is not fully understood; however, it is known that the organophosphorus molecules are efficient radical scavengers and flame quenching materials, and combustion processes are essentially exothermic free-radical reactions, so the existence of radical stabilizers impedes combustion by the quenching mechanism. On the other hand, the nitrogen-containing moieties release an inert gaseous byproduct to form a highly porous char that provides thermal insulation and prevents the combustion from spreading [23].

Phosphazene-based retardants as other retardants can be either additive or reactive. The most frequently additive modifiers contribute to the reduction of mechanical properties of resulting polymers forming a two-phase system that could be defective. Therefore, miscible or reactive flame retardants are preferable.

Our previous work [24] was devoted to a novel catalyst-flame retardant based on phosphazene for benzoxazine monomer based on bisphenol A, m-toluidine, and paraphormaldehyde (BA-mt). The efficiency of hexakis–(3–methylphenylamino)cyclotriphosphazene (PN-mt) as a catalyst and flame retardant was proved. In this work we intend to continue our study of this type of phosphazene catalyst-flame retardant, disclose the influence patterns of substituents in hexachlorocyclotriphosphazenes on the curing process, and give a brief study of thermal properties and flammability of the resulting polymer.

The following modifiers for BA-mt matrix were chosen: hexachlorocyclotriphosphazenes, Tetrakis–(3–methylphenylamino) dichlorocyclotriphosphazene, and hexakis–(3–methylphenylamino) cyclotriphosphazene and hexaphenoxycyclotriphosphazene. The first and the last are well-known compounds: an initial reagent and additive in flame retardant, respectively. They are expected to be the extreme points: the most reactive and nonreactive, respectively. The second and the third compounds, our novel modifiers, differed in the degree of m-toluidine substitution, so we expect the reduction of reactivity in the above mentioned list.

## 2. Materials and Methods

### 2.1. Starting Materials

Hexachlorocyclotriphosphazene (HCP) was purchased from Shandong Chuangyu Chemical Co., Ltd., Shandong, China. HCP was recrystallized from hexane before use. M-toluidine (Acros Organics, Geel, Belgium) was distilled twice under vacuum before use. Bisphenol A (4,4′–(Propane–2,2–diyl)diphenol) was supplied by by Kazanorgsintez PJSC, Kazan, Russia and used without further purification. Phenol was distilled before use. Excipients were used without preliminary purification. Solvents were purified according to known methods, and their physical characteristics corresponded to literature data [25].

### 2.2. Synthesis of Benzoxazine Monomer Based on Bisphenol A, M-Toluidine, and Paraphormaldehyde (BA-mt)

Benzoxazine BA-mt (bis(3–(m–tolyl)–3,4–dihydro–2H–1,3–benzoxazine) is a well-known commercialized monomer. It was chosen as the matrix for compositions to continue our study of a novel catalyst, revealed in our previous work [24]. It was synthesized using the method reported in the literature [26]. The product yield was 95%. Completely cured BA-mt has a glass transition temperature of 217 °C for samples cured under following conditions: 2 h 180 °C, 4 h 200 °C, and 1 h 210 °C.

### 2.3. Synthesis of Hexakis–(3–methylphenylamino)cyclotriphosphazene (PN-mt (6))

PN-mt (6) was obtained according to the method reported in our previous work [24]. A 250 mL round-bottom flask equipped with a magnetic stirrer and a reflux condenser was charged with 10 g (0.0287 mol) of HCP, dissolved in advance in 50 mL of 1,4-dioxane, 24.60 g (0.230 mol) of meta-toluidine, and 20.32 g (0.230 mol) triethylamine. The reaction mixture was refluxed under intensive stirring for 8 h. Further the solvent and excess of triethylamine and meta-toluidine were distilled off using a vacuum rotary evaporator. At first the remaining white mass with a yellowish tinge was repeatedly washed with distilled water. The product was twice precipitated from ethanol into water and, finally, dried in a vacuum until a constant mass was achieved. The final product was a white fine powder. The yield was 16.51 g (82%).

### 2.4. Synthesis of Tetrakis–(3–methylphenylamino)dichlorocyclotriphosphazene (PN-mt (4))

PN-mt (4) was obtained according to the following procedure. A 250 mL round-bottom flask equipped with a magnetic stirrer and a reflux condenser was charged with 10 g (0.0287 mol) of HCP, previously dissolved in 50 mL of 1,4-dioxane, 12.30 g (0.115 mol) of meta-toluidine and 11.61 g (0.115 mol) triethylamine. The reaction mixture was refluxed under intensive stirring for 6 h. Then, the solvent and excess of triethylamine and meta-toluidine were distilled off using a vacuum rotary evaporator. At first the remaining white mass with a yellowish tinge was repeatedly washed with distilled water. The product was twice reprecipitated from ethanol into water and, finally, dried in a vacuum until a constant mass was achieved. The final product was a white fine powder. The yield was 16.12 g (75%).

### 2.5. Synthesis of Hexaphenoxycyclotriphosphazene (HPP)

HPP was obtained according to the following procedure. A 200 mL round-bottom flask equipped with a reflux condenser and an overhead stirrer was charged with 10.00 g (0.0287 mol) HCP, 17.55 g (0.1868 mol) phenol, and 47.56 g (0.3446 mol) K_2_CO_3_, then 125 mL of acetonitrile was added. The mixture was boiled for 14 h. At the end of the process, the hot solution was filtered from salts and the solvent was distilled off. After that, the dry product was dissolved in 125 mL of toluene and washed three times with 10% sodium hydroxide solution. Then, it was washed with distilled water until neutral pH. The resulting solution was dried to remove traces of water with sodium sulfate for 1 h with intensive stirring. The resulting suspension was filtered, and the solvent was distilled off to constant weight. As a result, 16.32 g of white crystals of hexaphenoxycyclotriphosphazene were obtained. The yield was 82%.

### 2.6. Composition Preparation

In order to determine the effect of substituents in phosphazene compounds on the curing process of benzoxazine monomer BA-mt, the following formulations presented in Table 1 were prepared. The variation of modifier’s concentration in the mixtures was determined by several applied purposes: to achieve catalysis enough for compete cure under set curing conditions, to enhance flame resistance, and to maintain heat resistance at an appropriate level.

Compositions with HCP were prepared by dissolving of calculated amount of HCP in BA-mt melt at 100 °C for 10 min. The other compositions were prepared by dissolving the calculated amount of catalyst in 1,4-dioxane at 80 °C followed by addition of the obtained catalyst solution to the calculated amount of BA-mt. The mixtures were stirred at 80 °C for 10 min to achieve a homogeneous transparent solution. Subsequent degassing of the systems was performed at 120 °C for 30 min at a residual pressure of 1.0 kPa. Obtained compositions were either used as received for curing study or cured at 180 °C for 6 h for further thermal analysis and microscopy. Samples cured at 160 °C − 2 h + 180 °C − 2 h + 200 °C − 2 h + 210 °C − 1 h were utilized for flammability tests.

### 2.7. Measurements

The ^1^H, ^31^P, and ^13^C NMR spectra were obtained in DMSO-d6 solutions with a Bruker AV-400 spectrometer (Bruker Corporation, Bremen, Germany) operating at 400, 162, and 100 MHz, respectively. The signals, due to the deuterated solvents, were used as internal references. The chemical shifts of the signals were calculated relative to the signals of tetramethylsilane (^1^H, ^13^C) and phosphoric acid (^31^P), which were used as references. The spectra were processed with the help of the MestReNova Lab software package (version 12.0.4, MESTRELAB RESEARCH, S.L, Santiago de Compostela, Spain).

Differential scanning calorimeter DSC 214 Polyma (Netzsch, Selb, Germany) was used for monitoring thermal effects [27]. The temperature characteristics of the curing and glass transition temperatures of cured samples were determined according to ISO 11357-5:1999 [28] and ISO 11357-2:1999 [29], respectively. The heating rate for all measurements was 10 °C/min. All tests were performed in the temperature range 50–300 °C in a nitrogen atmosphere at a rate flow of 40 mL/min. The weight of the samples ranged from 5 to 10 mg. For data processing, Proteus Thermal Analysis version 8.0.2 software (Netzsch, Selb, Germany) was used.

The simultaneous thermal analyzer STA 449 F3 Jupiter (Netzsch, Selb, Germany) was used to evaluate the thermal stability of cured resin samples. The heating rate for all measurements was 20 °C/min. All tests were performed in the temperature range 50–1000 °C in a helium atmosphere at a flow rate of 100 mL/min. The weight of the samples ranged from 10 to 20 mg. For data processing, Proteus Thermal Analysis version 8.0.2. software (Netzsch, Selb, Germany) was used.

Flammability tests were carried out in accordance with Vertical Burning Test UL-94 (ASTM D3801–20a, Northbrook, IL, USA) [30] for 5 samples. The dimensions of the samples were 127 mm × 12.7 mm × 2 mm.

The morphology of cracks was investigated on an FEI Quanta 650 scanning electron microscope (Thermo Fisher Scientific, Waltham, MA, USA) in the secondary electron mode at an accelerating voltage of 10 kV. The phase structure was developed by plasma etching of a low-frequency oxygen discharge at a universal vacuum station (Edwards Coating System E306A, England). Sample preparation consisted of vacuum thermal sputtering of gold onto the crack surface (Edwards Coating System E306A, Burgess Hill, Great Britain).

## 3. Results and Discussions

### 3.1. Synthesis and Characteristics of Products

**Figure 2:**
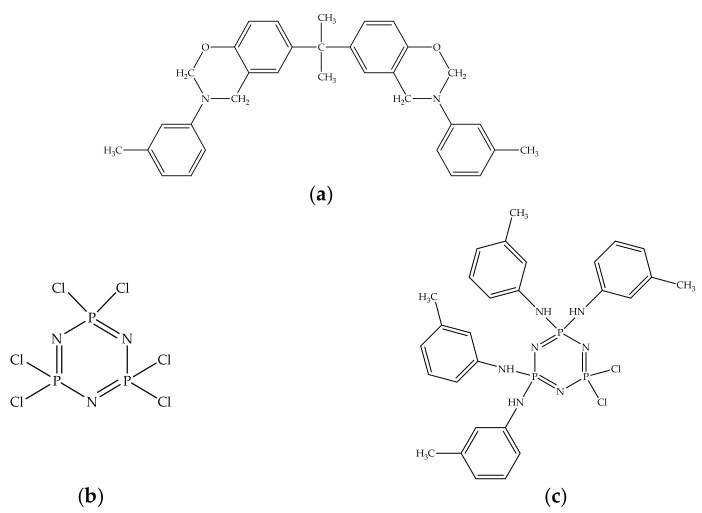

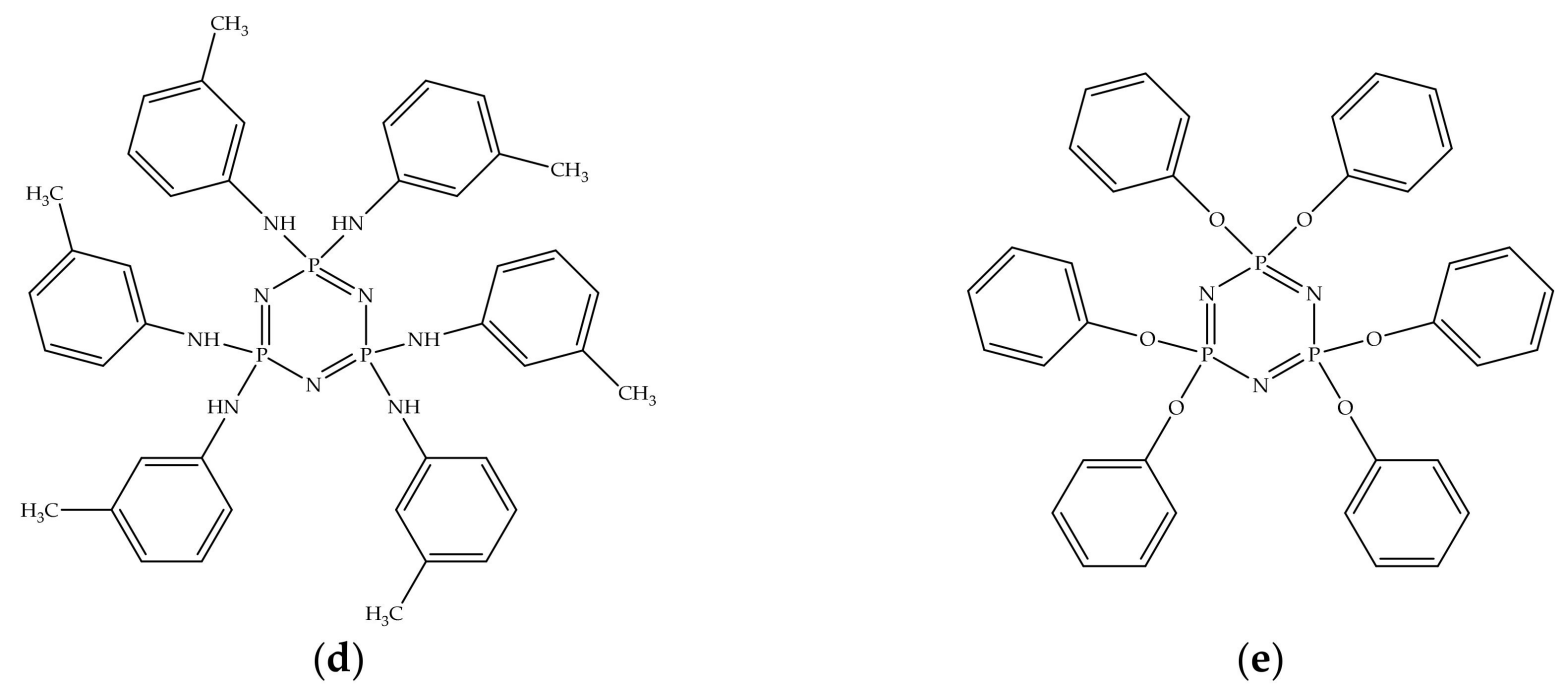
Structures of matrix BA-mt (**a**) and the modifiers: HCP (**b**), PN-mt(4) (**c**), PN-mt(6) (**d**), HPP (**e**).

Structures of all compounds used in compositions are presented in Figure 2.

Benzoxazine monomer BA-mt chosen for our catalysis study was obtained according to the method described in [26]. Monomer was characterized by ^1^H (Figure S1 in Supplementary Materials), ^13^C (Figure S2 in Supplementary Materials) NMR spectroscopy, DSC (Figure S3 in Supplementary Materials) and TGA (Figure S4 in Supplementary Materials). ^1^H NMR spectrum corresponds the structure of synthetized BA-mt. Signals at the area δ_H_ = 4.67 ppm (Ar–CH_2_–N) and δ_H_ = 5.42 ppm (O–CH_2_–N) correspond to methylene groups in the oxazine ring. The broadened signal at 4.25–4.5 ppm corresponds to the methylene protons of oligomeric compounds. The presence of oligomerized monomer in the product is either confirmed by a slight broadening of the DSC peak corresponded to the benzoxazine curing. Other thermal characteristics are T_peak_ = 233.3 °C and ΔH = 320.1 J/g. Thermal resistance of the cured matrix was evaluated by TGA. The temperature of 5% mass loss was 358.5 °C and residual mass at 1000 °C was 30.38%.

The modifier HPP obtained according to Section 2.5 was characterized by ^1^H (Figure S5 in Supplementary Materials), ^13^C (Figure S6 in Supplementary Materials), and ^31^P (Figure S7 in Supplementary Materials) NMR spectroscopy, DSC (Figure S8 in Supplementary Materials) and TGA (Figure S9 in Supplementary Materials). The ^1^H-NMR spectrum of the product shows proton signals in the region of 6.85–7.40 ppm that correspond to signals of phenoxy substituents. In the area of 2.5 ppm there is a signal of residual protons of DMSO-d6. The ^13^C-NMR spectrum contains signals of carbon atoms of benzene rings of phenoxy substituents in the ranges 120.48–129.90 ppm and 149.92 ppm. In the region 39.13–39.97 ppm carbon signal of DMSO-d6 is present. The ^31^P-NMR spectrum contains signals of phosphorus atoms in HPP (8.55 ppm). DSC exotherm detected the melting temperature of 112.6 °C. Destruction of the compound was evaluated by TGA revealing the temperature of 5% mass loss being 324.8 °C and residual mass at 1000 °C − 4%.

The modifier PN-mt(4) obtained according to Section 2.4 was characterized by ^1^H (Figure S10 in Supplementary Materials), ^13^C (Figure S11 in Supplementary Materials), and ^31^P (Figure S12 in Supplementary Materials) NMR spectroscopy, DSC (Figure S13 in Supplementary Materials) and TGA (Figure S14 in Supplementary Materials). The ^1^H-NMR spectrum of the product shows proton signals in the range of 2.12–2.24 ppm which correspond to the signals of the methyl groups of m-toluidine, 7.02–7.08 ppm and 8.02–8.05 ppm the protons of the benzene ring of m-toluidine, as well as 6.64–6.67 ppm corresponding signals of protons of secondary amine groups attached to phosphorus atoms. The ratio of protons of the secondary amine groups to methyl protons of m-toluidine is 1:3 as calculated. The ^13^C-NMR spectrum of the product contains signals from the carbon atoms of the methyl groups of m-toluidine in the regions of 21.19–21.28 ppm, 115.02–141.37 ppm atoms of benzene rings. In the range 38.85−40.11 ppm signals of carbons ^13^C DMSO-d6 are present. The ^31^P-NMR spectrum of the product contains signals of phosphorus atoms corresponding to the degrees of substitution: three- (geminal)—in the regions of 22–23, 6.5–7.5, and −0.5–0.5 ppm, and tetra (geminal) −21–22 and (−1.5)–(−1) ppm. Such a specific nature of substitution, which differs from the most common non-geminal one, requires a separate study. DSC exotherm detected the melting temperature of 195.2 °C. Destruction of the compound was evaluated by TGA revealing the temperature of 5% mass loss being 261.9 °C and residual mass at 1000 °C − 35%.

The modifier PN-mt(6) obtained according to Section 2.3 was characterized by ^1^H (Figure S15 in Supplementary Materials), ^13^C (Figure S16 in Supplementary Materials), and ^31^P (Figure S17 in Supplementary Materials) NMR spectroscopy, DSC (Figure S18 in Supplementary Materials) and TGA (Figure S19 in Supplementary Materials). The 1H-NMR spectrum of the product shows proton signals in the range of 2.29 ppm which correspond to the signals of the methyl groups of m-toluidine, and 6.66–7.32 ppm—to the protons of the benzene ring of m-toluidine. It was considered that the signal of the proton of the amine group was superimposed on the signal of one of the protons of the aromatic ring, as reported in the article [24]. The absence of a similar overlap of signals for PN-mt(4) could be explained by the fact that, in the case of incomplete substitution of chlorine atoms in the initial HCP, the fraction of intermolecular hydrogen bonding increased (between the molecules of PN-mt(4) and the solvent, DMSO-d6). Therefore, the protons of the secondary -NH- groups in PN-mt (4) were more deshielded and shifted to the downfield compared with PN-mt(6). The ^13^C-NMR spectrum of the product contains signals from the carbon atoms of the methyl groups of m-toluidine in the region of 21.39 ppm, signals in the region of 114.87–142.56 ppm. correspond to the atoms of the benzene rings. In the region 39.08–39.91 ppm signals of carbon ^13^C DMSO-d6 are present. The ^31^P-NMR spectrum of the product contains a singlet signal in the region of 2.39 ppm, which indicates the complete replacement of chlorine atoms in the initial HCP by meta-toluidine radicals. DSC exotherm detected the melting temperature of 242.7 °C. Destruction of the compound was evaluated by TGA revealing the temperature of 5% mass loss being 266.7 °C and residual mass at 1000 °C − 30.74%.

Recrystallized HCP was characterized by ^31^P NMR- spectrum as well as by DSC and TGA presented in Supplementary Figures S20, S21, and S22, respectively. The ^31^P-NMR spectrum contains singlet with δ_P_ = 19.9 ppm of phosphorus atom. DSC exotherm detected the melting temperature of 112.7 °C. According to the TG analysis the sublimation of HCP is observed [31].

### 3.2. The Influence of Phosphazene Compounds on the Curing Process

**Figure 3:**
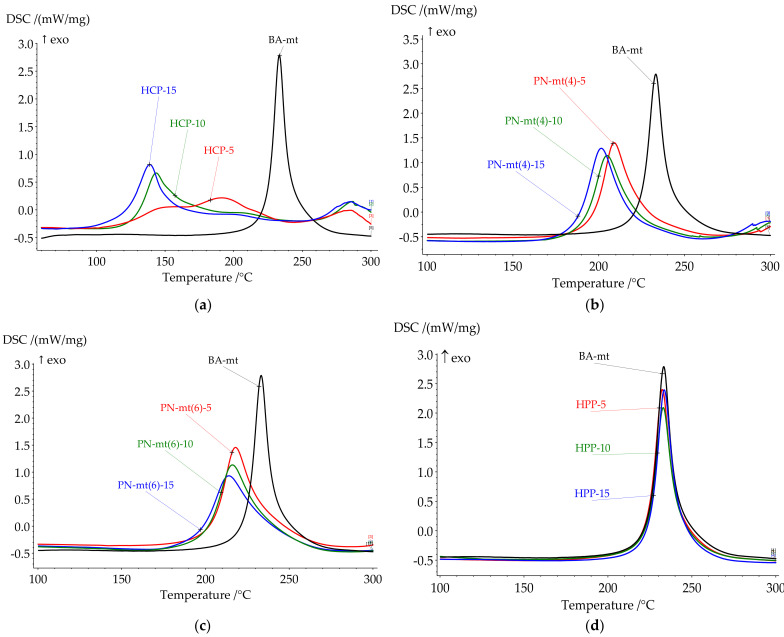
DSC curves of uncured compositions with HCP (**a**), PN-mt(4) (**b**), PN-mt(6) (**c**), HPP (**d**).

The influence of chosen modifiers on the curing process was studied by DSC method. The key points of DSC scans are presented in Table 2. All DSC curves are presented in Figure 3.

HPP almost does not change key temperatures, but influences the heat released. Nevertheless, the lone electron pair of the nitrogen atoms in PN-mt located in the phosphazene core was expected to catalyze benzoxazine ring-opening polymerization (ROP), no catalysis was observed.

**Figure 4:**
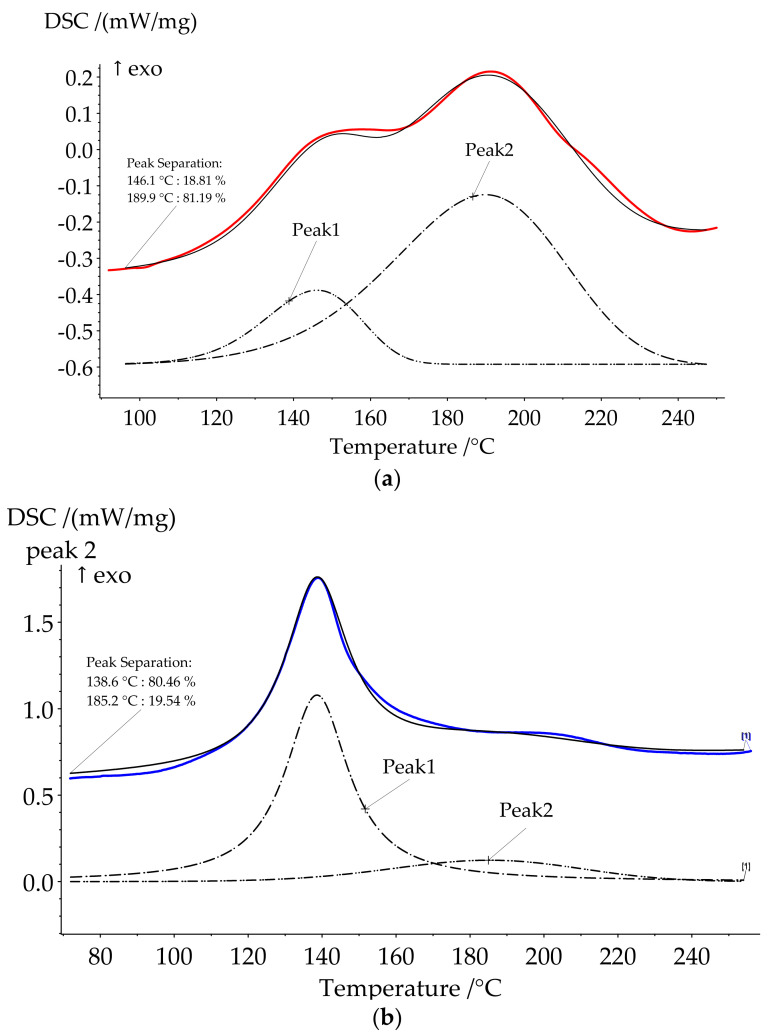
Peak separation for formulations HCP-5 (**a**) and HCP-15 (**b**).

**Figure 5:**
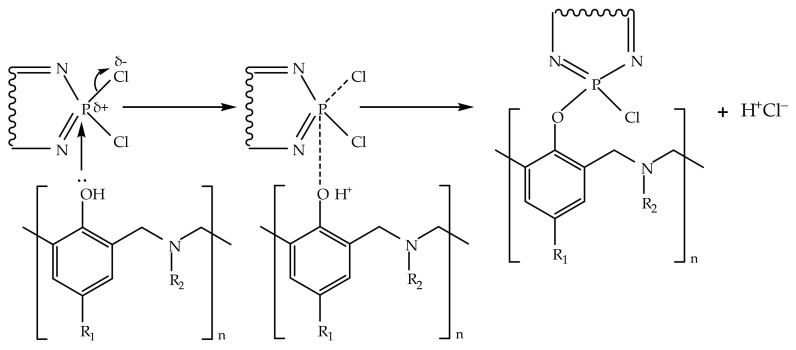
The proposed mechanism of phenolysis of chlorocyclophosphazenes.

**Figure 6:**
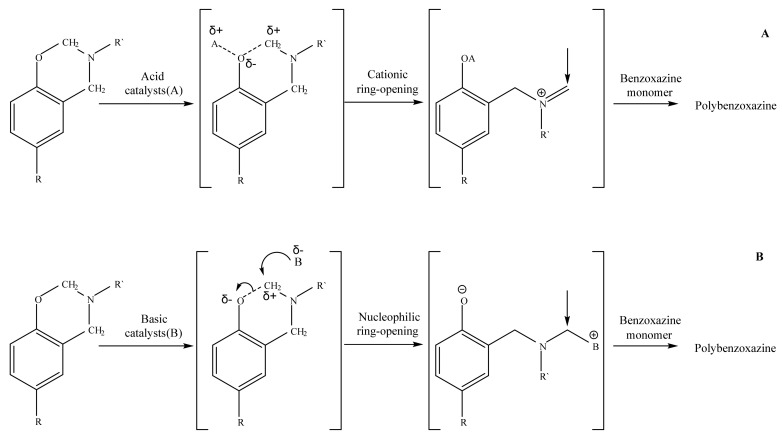
The proposed cationic catalytic mechanism ((**A**)—in case of released HCl) and anionic catalytic mechanism ((**B**)—in case of secondary amine group).

The use of HCP as a catalyst of benzoxazine curing significantly decreased the onset temperature of the reaction. The curing process of compositions was characterized by complex process with at least two stages. That is why peak separation was carried out (Figure 4). The ratio between the first and second peaks areas increased in a row, HCP-5, HCP-10, and HCP-15 being 19:81, 72:28, and 81:19. We suppose that oligomeric compounds presented in BA-mt resin react by nucleophilic substitution with HCP (Figure 5). The released HCl catalyzed ROP corresponded to the first peak (Figure 6a). With the temperature raise the autocatalytic effect became more significant associated with the increase of hydroxylic group’s concentration during ROP. Further curing of composition led to decrease in mobility of molecules and decrease in HCl concentration due to steric hindrance or the fixation of HCl by tertiary nitrogen atoms in the formed Mannich bridges. This effect resulted in the second wide peak on DSC curve corresponded to homopolymerization of benzoxazine. With an increase of HCP content, the first stage prevails over the second accompanying by the growth of heat released.

The most interesting catalytic effect was observed in compositions with PN-mt(4) and PN-mt(6). The addition of 5 phr PN-mt(4) caused the decrease of the onset temperature to 196.3 °C; the addition of 5 phr PN-mt(6) decreased the onset temperature to 203.5 °C. The further increase of catalyst containment to 15 phr slightly reduces onset temperature (to 186.7 °C in case of 15 phr PN-mt(4) and to 196.5 °C in case of 15 phr PN-mt(6)). Additionally catalysts affect the amount of released heat. In case when we used 5 phr PN-mt(4) released heat decreases to 280.0 J/g; 5 phr PN-mt(6)—released heat decreases to 312.3 J/g. Further increase of PN-mt(4) content did not significantly change the exothermic effect of the polymerization. The unique catalytic effect consisted in the possibility to catalyze benzoxazine ROP by released HCl for PN-mt(4) (Figure 6a) and a nucleophilic attack of the lone electron pair of the nitrogen atoms in PN-mt located in substituents (Figure 6b) on the methylene bridge connecting the oxygen and nitrogen atoms in the benzoxazine ring for PN-mt(4) and PN-mt(6) [32]. The basic catalytic effect was weaker for the benzoxazine polymerization than acidic one. Thus, the use of PN-mt(4) led to the implementation of two different mechanisms of catalysis resulting in a greater catalytic effect than in case of PN-mt(6). Moreover we supposed that compositions with PN-mt(6) were induced by steric hindrance.

Thus, the catalytic activity of obtained phosphazene compounds on curing process of benzoxazine resin decreased in row: HCP < PN-mt(4) < PN-mt(6) < HPP. This tendency was also confirmed by the fact that acidic catalysis was more effective than basic one. The absence of catalytic effect of HPP proved that there was no catalytic activity of lone electron pair of nitrogen atom in the core of phosphazene ring.

In comparison with literature data, the catalytic effect of HCP was quite explicit. The lowering of the onset by 100 °C was a significant effect, which was better than the catalytic effect of acids and phenols described in [8]. The decrease of peak polymerization temperature for PN-mt(6) and PN-mt(4) was 15 °C and 24 °C respectively. This effect was in good agreement with literature data of catalytic effect of amines, imidazoles, and indole on benzoxazine ring-opening reaction. Obtained phosphazene compounds were secondary amines and the catalytic effect was similar to other secondary amines [33].

### 3.3. Thermal Analysys of Cured Compositions

**Figure 7:**
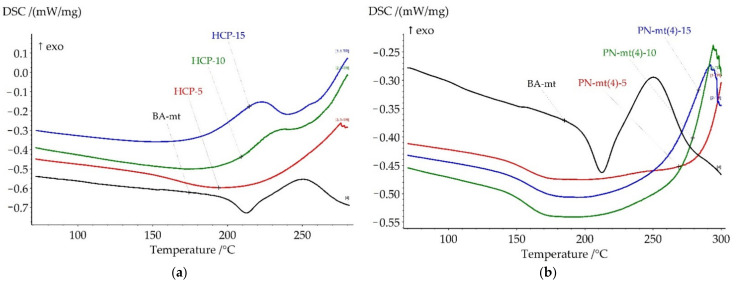

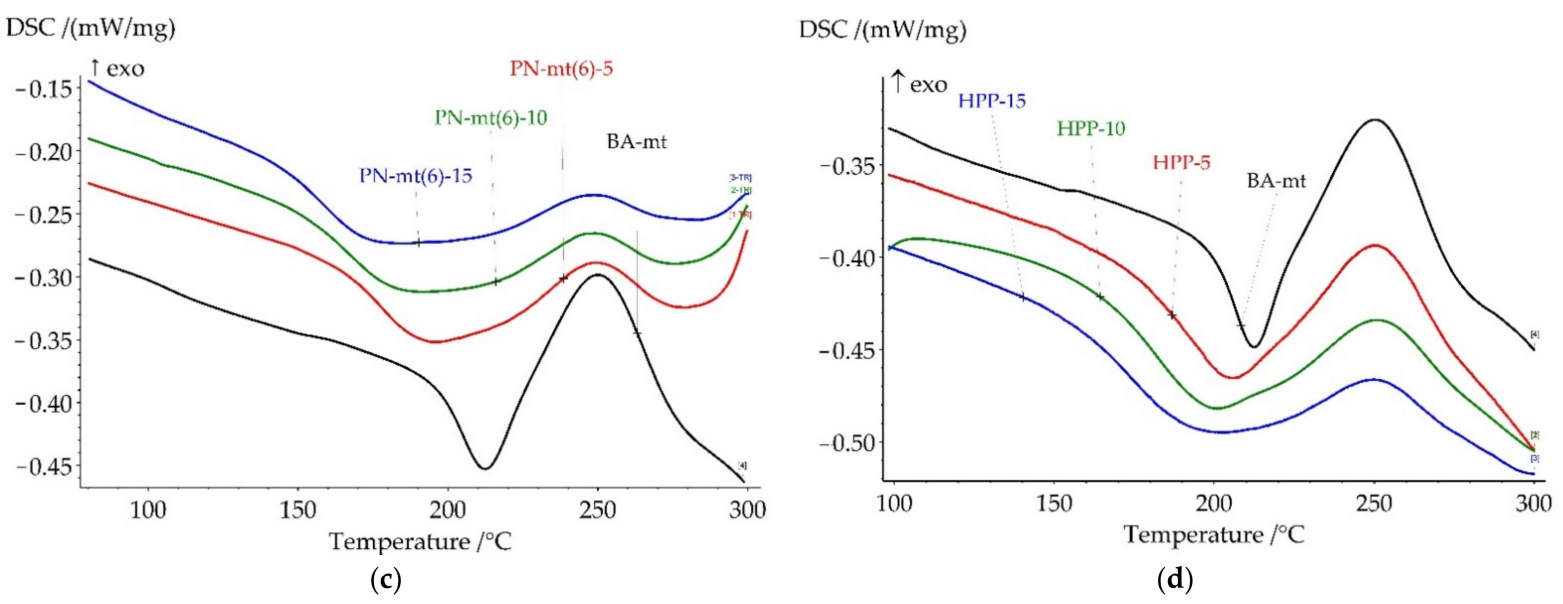
DSC curves of cured compositions with HCP (**a**), PN-mt(4) (**b**), PN-mt(6) (**c**), HPP (**d**) in the scanning mode.

**Figure 8:**
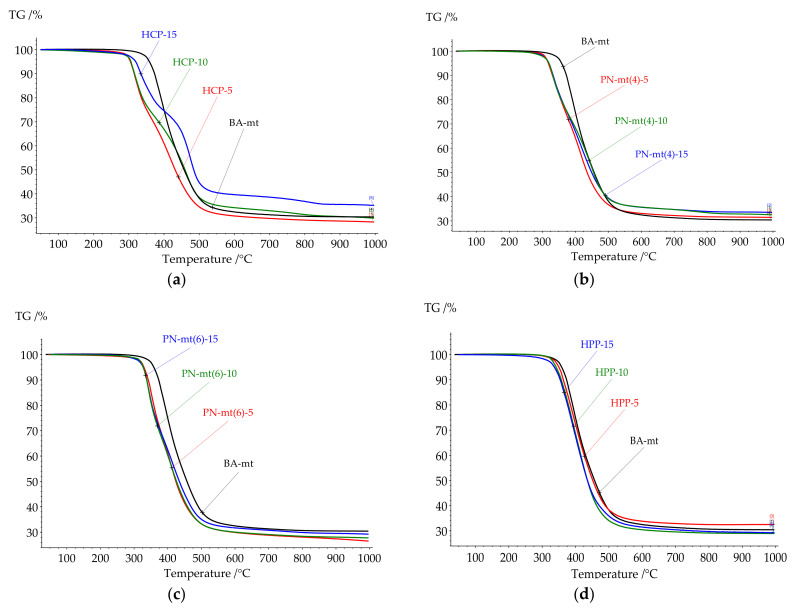
TGA curves of cured compositions with HCP (**a**), PN-mt(4) (**b**), PN-mt(6) (**c**), HPP (**d**) in the scanning mode.

All compositions under study were cured according to the modeling curing program 180 °C for 6 h. Thermal analysis of resulting polymers was proceeded by DSC and TGA methods to correlate heat flow effects with mass loss caused by destruction. The key points of DSC and TGA results were presented in Table 3. All DSC curves were presented in Figure 7 and TGA curves were presented in Figure 8.

Glass transition temperature (Tg) of the neat BA-mt cured at 180 °C for 6 h was 203.3°C. After glass transition the exothermic peak was observed with enthalpy 38.4 J/g corresponding to the post-curing of benzoxazine resin. This temperature program resulted in incomplete curing for matrix (degree of conversion 88%) which was chosen especially for explicit evaluation of modifiers influence in compositions on the final degree of conversion.

Cured compositions with HCP addition did not demonstrate glass transition until a significant exothermic effect (Figure 7a). According to the DSC analysis the exothermic effect starts for HCP-5 at 215 °C; for HCP-10 at 210 °C; and for HCP-15 at 200 °C. According to the TG analysis the weight loss started at 300 °C so we supposed that exothermic effect was related to the post-curing reaction and the begin of destruction. The second DSC scan confirmed our assumption as no exothermic peaks until 280 °C were observed (Figure S23 in Supplementary Materials).

The addition of PN-mt(4) led to the complete cure, while PN-mt(6) and HPP led to the slight increase of degree of conversion: 98% and 97% respectively for 15 phr compared with neat BA-mt (88%). The addition of 5 phr of HCP and PN-mt(4) dramatically reduced Tg of polymers till 161.4 °C and 158.5 °C, respectively. Further raise of concentration of HCP and PN-mt(4) till 15 phr had different effects. HCP caused the great increase of Tg to 221.7 °C, while composition PN-mt(4)-15 reached only 160.5 °C. PN-mt(6) and HPP influenced in another way. They monotonously reduced Tg from 180.5 °C and 195.9 °C for 5 phr to 160.0 °C and 176.3 °C for 15 phr, respectively.

The outstanding dependence of glass transition temperature for HCP could be related to the formation of rigid polymer network due to high reactivity of HCP detected by DSC (Figure 3a). One of the indirect evidences for proposed curing mechanism of phenolysis (Figure 5) was the rust on the steel casting mold which was used for HCP curing.

The other modifiers reduce glass transition temperature. This effect could be caused by incomplete compatibility of resin and catalyst. Another reason was the significant size of the modifier molecules, which was comparable to nanoparticles with a diameter of 1–2 nm. It resulted in a change in the spatial regularity of the formed polybenzoxazine network and a decrease in the number of hydrogen bonds between hydrogen atoms in phenolic groups and nitrogen in polymerized benzoxazines. The decrease of glass transition temperature upon addition of PN-mt(4) could also be explained by the fact that the nucleophilic substitution reaction was incomplete and chlorine atoms of PN-mt(4) were not completely replaced by benzoxazine polymeric matrix. Based on this assumption PN-mt(4) participated in the reaction as monofunctional additive disrupted the organized benzoxazine network resulted in decreasing of glass transition temperature.

DSC analysis revealed that for compositions with HCP, PN-mt(4) and PN-mt(6) at the end of a measurement an exothermic peak was observed that indicated that samples lost their thermal stability. To study degradation process TGA measurements in inert atmosphere were carried out.

Thermal stability of the studied compositions had the same tendencies as the glass transition temperature. The addition of HCP and PN-mt(4) dramatically reduced the onset of thermal destruction followed by a slight increase of values in the row from 5 to 15 phr. PN-mt(6) and HPP influenced in another way. They monotonously reduced onset of the destruction.

It was proposed in literature that the thermal decomposition of polybenzoxazines occurs stepwise [34,35]. At the first stage of the destruction, aromatic compounds were formed (benzene, derivatives of phenol, aniline). On the second step low-molecular compounds (hydrocarbons, carbon dioxide, aliphatic amines, etc.) were formed, followed by carbonization.

Cured compositions with HCP, PN-mt4, and PN-mt6 demonstrated similar thermal characteristics. The destruction of cured compositions endured two stages of decomposition. Whereas in systems with HPP only one stage was observed.

### 3.4. Morphology of Resulting Polybenzoxazines

**Figure 9:**
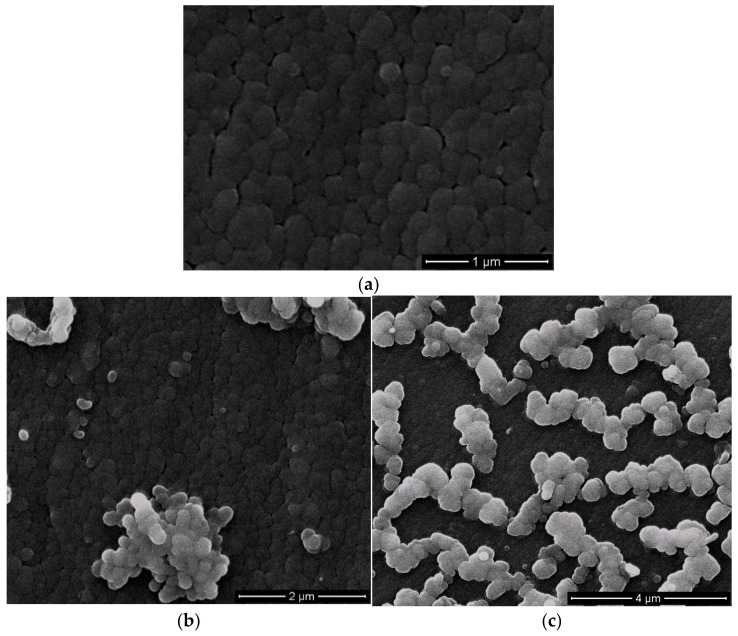

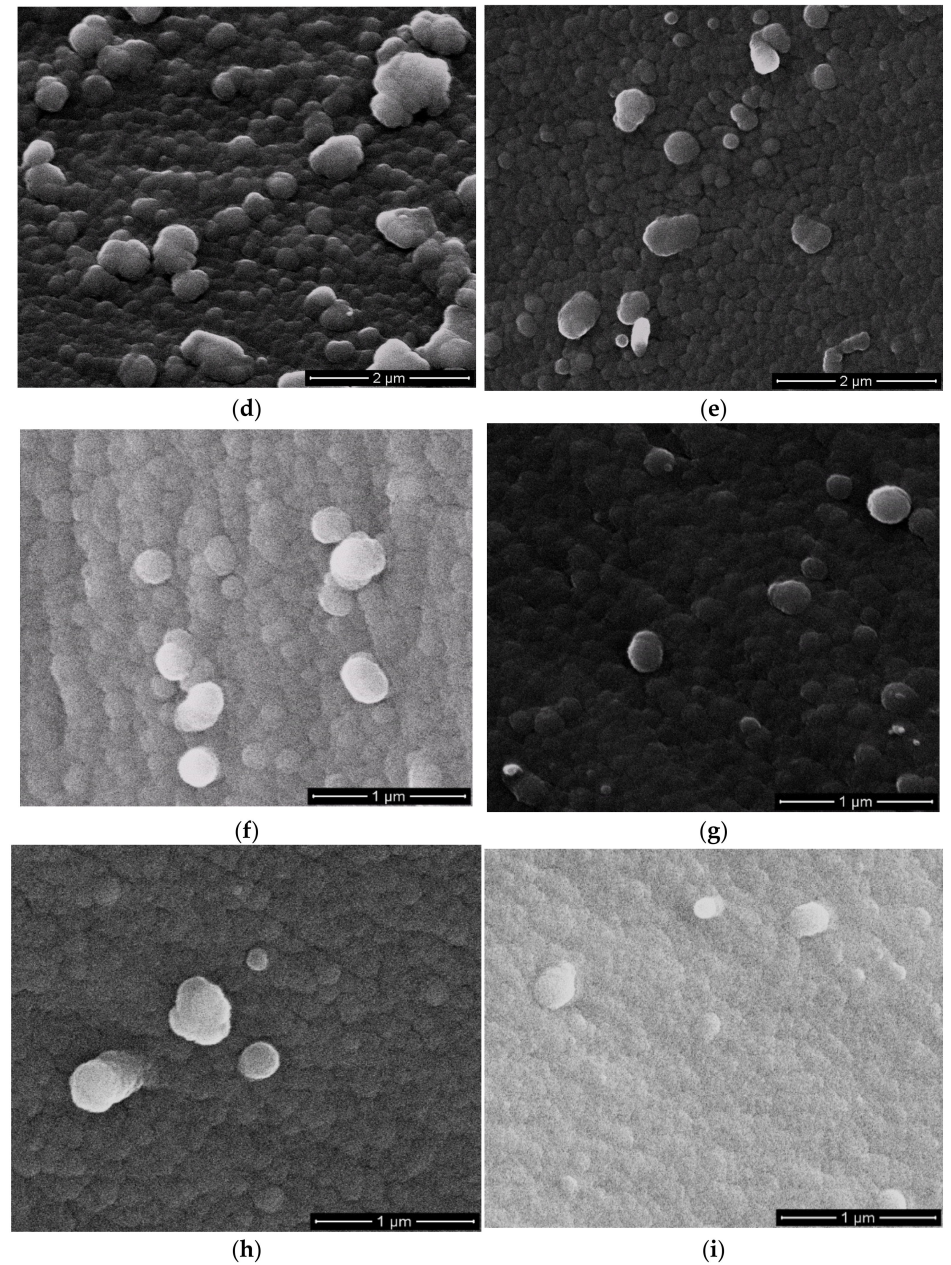
Morphology of cured BA-mt (**a**) and compositions HCP-5 (**b**), HCP-15 (**c**), PN-mt(4)-5 (**d**), PN-mt(4)-15 (**e**), PN-mt(6)-5 (**f**), PN-mt(6)-15 (**g**), HPP-5 (**h**), HPP-15 (**i**).

It was important for the flame retardant additive and catalysts to be dispersed or miscible in the polymer matrix for improved properties. The morphology of cured compositions was studied by scanning electron microscopy (SEM). The structure of cured neat BA-mt (Figure 9a) revealed homogeneous globular structure. Cured compositions with additives was characterized by matrix-dispersion phase structure (Figure 9b–i). The matrix consists of globular phase with the size of 20–100 nm. The dispersed phase was irregularly distributed in the matrix and consists of globules with the size of 130–350 nm. In some cases, the globules of dispersed phase formed associates. This phase structure was typical for all systems under study regardless of reactivity of additives and their concentration. We supposed that forming of dispersed phase structure occurred during polymerization which led to deterioration in thermodynamic compatibility of phases. As a result the heterogeneous phase structure was formed. For the system HPP-15 one could see minor amount of the second phase, so mostly this system was compatible, and its additive influenced greatly on Tg. It is well known that there are two main factors that influence Tg. The first one is compatibility of matrix and modifier. If the system is compatible its glass transition temperature would shift to the glass transition temperature of modifier. The second factor is the influence of modifier on the network density due to copolymerization of matrix and modifier. Thus glass transition temperature of systems with HCP, PN-mt(4), and PN-mt(6) was deendent on the predominance of one of these factors.

### 3.5. Flammability of Cured Compositions

Flammability of cured compositions was estimated by UL-94 standard. The results of burning tests are shown in Table 4. The cured neat BA-mt achieved only V-1 flammability category of the UL-94 standard. The use of obtained additives in amount of 5 phr does not reduce the total burning time of the specimens except for the use of HCP. This effect could be explained by low concentration of the additive and also by the introduction of a phosphazene ring to the polymeric matrix in case of using HCP. For all used flame retardant agents, the total time of burning decreased with the increase of additive concentration due to an increase of the phosphazene containment. It is also important to note that no afterglow, either of burning particles or drops, was observed during burning tests. The HPP concentration more than 10 phr allows to achieve the V-0 flammability category. The HPP is a well-known commercialized flame retardant which efficiency was proved by many works [22,36,37]. HCP affects the flammability of benzoxazine resin at a content of 5 phr and more. The concentration of 10 phr HCP and more resulted in plastic with V-0 flammability category. We supposed that the great flame-retardant properties of HCP were associated with introducing the phosphazene ring to polymeric matrix and high phosphorous content due to low molecular mass of HCP (versus the other additives). We supposed that the flame retardant effect of PN-mt(4) and PN-mt(6) will be similar to the HPP. However, the total time of burning for compositions containing PN-mt(4) and PN-mt(6) is higher than for compositions with HPP. PN-mt(4) contained less phenyl residues resulted in better flammability of compositions with PN-mt(4). Another possible explanation is that PN-mt(4) and PN-mt(6) affected the polymerization process and hence could affect the structure of the matrix. The composition with 15 phr PN-mt(6) achieved the V-0 flammability category but due to the above-mentioned reasons compositions with PN-mt(4) reaches only V-1 flammability category.

According to calculationos by the Van Krevelen–Hovtyzer equation LOI all polymer compositions could be related to nonflammable category as LOI > 28 [38]. So, we can conclude that all presented additives improve the flame resistance of benzoxazine resin due to an increase of phenyl residues content and to the phosphazene structure that possesses the synergistic effect of phosphorus and nitrogen introduced into the mixture.

The addition of phosphazene compounds also affect the flammability of benzoxazine resin. It was shown that 5 phr of all catalysts except HCP did not decrease the burning time of plastic specimens. HCP, PN-mt(6), and HPP allowed to reach the V-0 flammability category when 15 phr was added. However, the addition of novel additive PN-mt(4) to benzoxazine resin did not reach the V-0 category but decreased the total burning time from 131 s (0 phr) to 71 s (15 phr).

## 4. Conclusions

The influence of modifiers with substituents in the phosphazene core including novel flame retardant agent–catalyst PN-mt(4) on the curing process, thermal stability, morphology, and flammability of benzoxazine resin BA-mt was studied in this work. The catalytic activity of phosphazene compounds on the curing process of benzoxazine resin decreased in the row: HCP > PN-mt(4) > PN-mt(6) > HPP where HPP was totally inactive. HCP, PN-mt(4), and PN-mt(6) catalysts decreased the onset polymerization temperature and residual enthalpy for cured samples. The two catalysis mechanisms were proposed. For chlorine-containing catalysts HCl released during phenolysis catalyze the ROP by acidic mechanism proposed in our work. For m-toluidine containing catalysts the lone electron pair of the nitrogen atoms located in substituents catalyze ROP by basic mechanism. The addition of minor amounts of modifiers overall caused the decrease of the glass temperature and the thermal stability of the cured resin. However, the addition of 15 phr HCP resulted in the rigid polymer network due to multifunctionality of modifier and hence increased the glass transition temperature. The morphology of cured modified compositions was characterized by matrix-dispersion phase structure. The use of phosphazene catalysts affects the flammability of benzoxazine resin. Incorporation of HCP, PN-mt(6), and HPP allowed to reach the V-0 flammability category when 15 phr was added.

## Figures and Tables

**Table 1:** Formulations of mixtures in parts per hundred resin (phr).

Formulation Index	Catalyst, Phr	Catalyst
BA-mt ^1^	0	−
HCP-5	5	HCP ^2^
HCP-10	10	
HCP-15	15	
PN-mt (4)-5	5	PN-mt (4) ^3^
PN-mt (4)-10	10	
PN-mt (4)-15	15	
PN-mt (6)-5	5	PN-mt (6) ^4^
PN-mt (6)-10	10	
PN-mt (6)-15	15	
HPP-5	5	HPP ^5^
HPP-10	10	
HPP-15	15	

^1^ BA-mt—bis(3—(m—tolyl)—3,4-dihydro—2H—1,3-benzoxazine, ^2^ HCP-Hexachlorocyclotriphosphazene, ^3^ PN-mt(4)—Tetrakis-(3-methylphenylamino)dichlorocyclotriphosphazene, ^4^ PN-mt(6)—Hexakis-(3-methylphenylamino)cyclotriphosphazene, ^5^ HPP—Hexaphenoxycyclotriphosphazene.

**Table 2:** Characteristic temperatures of DSC curves of uncured samples.

Formulation Index	T_onset_, °C	T_peak_, °C	T_end_, °C	ΔH, J/g
BA-mt	224.6	233.3	241.8	320.1
HCP-5	121.7 (142.1) *	146.1 (189.9) *	167.5 (227.6) *	208.6
HCP-10	122.4 (139.2) *	143.6 (187.1) *	167.9 (225.8) *	215.6
HCP-15	121.8 (134.7) *	138.6 (185.1) *	155.5 (224.8)	225.1
PN-mt(4)-5	196.4	209.0	225.3	280.0
PN-mt(4)-10	190.6	204.9	222.0	277.2
PN-mt(4)-15	186.7	201.6	218.8	269.4
PN-mt(6)-5	203.5	218.0	236.2	312.3
PN-mt(6)-10	200.4	216.0	238.2	301.4
PN-mt(6)-15	196.5	214.0	242.3	285.6
HPP-5	223.4	232.4	243.1	290.6
HPP-10	223.7	233.0	243.1	261.8
HPP-15	224.7	233.5	242.1	254.3

* the second peak according to the peak separation (Figure 4).

**Table 3:** Characteristic temperatures of TGA, DSC and LOI data of cured samples and TGA data of neat modifiers.

Formulation Index	TGA			DSC		LOI **
	T_onset_, °C	T_5%_, °C	Residual Mass, %	T_g(middle)_, °C	ΔH_res_, J/g	
BA-mt	348.5	358.5	30.38	203.3	38.4	29.652
HCP-5	298.7	304.7	28.27	161.4 *	0 *	28.808
HCP-10	299.6	306.1	29.88	193.4 *	0 *	29.452
HCP-15	312.5	319.6	35.22	221.7 *	0 *	31.588
PN-mt(4)-5	307.2	318.3	31.43	158.5	1.0	30.072
PN-mt(4)-10	313.1	320.0	32.51	159.3	0	30.504
PN-mt(4)-15	314.4	320.3	33.49	160.5	0	30.896
PN-mt(6)-5	329.1	329.2	26.49	180.5	9.3	28.096
PN-mt(6)-10	324.8	327.1	27.74	167.4	6.0	28.596
PN-mt(6)-15	322.9	327.0	29.20	160.0	5.0	29.18
HPP-5	345.9	350.1	32.52	195.9	21.7	30.508
HPP-10	342.6	341.9	30.91	186.5	14.4	29.864
HPP-15	336.5	336.5	29.20	176.3	9.1	29.18
HCP	118.5	−	4.56	−	−	
PN-mt(4)	257.6	−	34.82	−	−	
PN-mt(6)	254.1	−	30.74	−	−	
HPP	332.0	−	3.90	−	−	

* DSC results after the second run (Figure S23 in Supplementary Materials). ** Char yield at 1000 °C values were used for calculation.

**Table 4:** Flammability test results according to UL-94 standard.

Composition	Flame Application	Specimen № 1	Specimen № 2	Specimen № 3	Specimen № 4	Specimen № 5	ΣT, S	Afterglow, S	Drops	Category
BA-mt	*t*_1_, *s*	24	10	3	12	22	131	no	no	V-1
	*t*_2_, *s*	3	4	21	30	2				
HCP-5	*t*_1_, *s*	20	17	8	5	21	95	no	no	V-1
	*t*_2_, *s*	7	2	0	13	2				
HCP-10	*t*_1_, *s*	0	0	0	1	0	20	no	no	V-0
	*t*_2_, *s*	0	8	6	0	5				
HCP-15	*t*_1_, *s*	3	0	0	0	0	8	no	no	V-0
	*t*_2_, *s*	0	3	0	0	2				
PN-mt(4)-5	*t*_1_, *s*	24	23	20	25	19	131	no	no	V-1
	*t*_2_, *s*	8	6	0	1	5				
PN-mt(4)-10	*t*_1_, *s*	17	25	7	9	26	127	no	no	V-1
	*t*_2_, *s*	10	5	15	6	7				
PN-mt(4)-15	*t*_1_, *s*	15	12	8	7	11	72	no	no	V-1
	*t*_2_, *s*	4	2	0	11	2				
PN-mt(6)-5	*t*_1_, *s*	12	21	24	17	20	135	no	no	V-1
	*t*_2_, *s*	14	2	10	9	6				
PN-mt(6)-10	*t*_1_, *s*	20	29	25	16	19	122	no	no	V-1
	*t*_2_, *s*	2	0	4	3	4				
PN-mt(6)-15	*t*_1_, *s*	7	1	1	2	4	39	no	no	V-0
	*t*_2_, *s*	2	5	8	9	0				
HPP-5	*t*_1_, *s*	0	9	0	19	4	134	no	no	V-1
	*t*_2_, *s*	12	18	20	27	25				
HPP-10	*t*_1_, *s*	1	1	0	1	0	15	no	no	V-0
	*t*_2_, *s*	1	2	1	7	1				
HPP-15	*t*_1_, *s*	0	1	0	0	1	4	no	no	V-0
	*t*_2_, *s*	0	0	0	2	0				

## Data Availability

The data presented in this study are available on request from the corresponding author.

## Supplementary Materials

The following are available online at https://www.mdpi.com/article/10.3390/polym13183111/s1, Figure S1: 1H NMR spectrum of benzoxazine BA-mt. Figure S2: 13C NMR spectrum of benzoxazine BA-mt. Figure S3. DSC curve of BA-mt. Figure S4. TGA curve of BA-mt. Figure S5. 1H NMR spectrum of HPP. Figure S6. 13C NMR spectrum of HPP. Figure S7. 31P NMR spectrum of HPP. Figure S8. DSC curve of HPP. Figure S9. TGA curve of HPP. Figure S10. 1H NMR spectrum of PN-mt(4). Figure S11. 13C NMR spectrum of PN-mt(4). Figure S12. 31P NMR spectrum of PN-mt(4). Figure S13. DSC curve of PN-mt(4). Figure S14. TGA curve of PN-mt(4). Figure S15. 1H NMR spectrum of PN-mt(6). Figure S16. 13C NMR spectrum of PN-mt(6). Figure S17. 31P NMR spectrum of PN-mt(6). Figure S18. DSC curve of PN-mt(6). Figure S19. TGA curve of PN-mt(6). Figure S20. 31P NMR spectrum of HCP. Figure S21. DSC curve of HCP. Figure S22. TGA curve of HCP. Figure S23. DSC curves of the second DSC run of cured compositions with HCP.
